# Supplementary material for: Pyruvate Dehydrogenase Kinase 1 inhibition mediated oxidative phosphorylation enhancement in cartilage promotes osteoarthritis progression
Source: BMC Musculoskelet Disord. 2023 Jul 20;24:597. doi: 10.1186/s12891-023-06585-6 (PMC10357736; doi:10.1186/s12891-023-06585-6)
Supplement: Supplementary file 1 — Additional file 1. [file 12891_2023_6585_MOESM1_ESM.zip › BP.Gsea.1653623667859/GOBP_RELEASE_OF_CYTOCHROME_C_FROM_MITOCHONDRIA.html]

Details for gene set GOBP\_RELEASE\_OF\_CYTOCHROME\_C\_FROM\_MITOCHONDRIA[GSEA]

|  || Dataset | 55457\_collapsed\_to\_symbols.cls.cls #OA\_versus\_normal.cls.cls #OA\_versus\_normal\_repos |
| Phenotype | cls.cls#OA\_versus\_normal\_repos |
| Upregulated in class | OA |
| GeneSet | GOBP\_RELEASE\_OF\_CYTOCHROME\_C\_FROM\_MITOCHONDRIA |
| Enrichment Score (ES) | 0.36521202 |
| Normalized Enrichment Score (NES) | 1.4733148 |
| Nominal p-value | 0.025906736 |
| FDR q-value | 0.38613555 |
| FWER p-Value | 1.0 |
Table: GSEA Results Summary

  

Fig 1: Enrichment plot: GOBP\_RELEASE\_OF\_CYTOCHROME\_C\_FROM\_MITOCHONDRIA      
 Profile of the Running ES Score & Positions of GeneSet Members on the Rank Ordered List

  

| SYMBOL | TITLE | RANK IN GENE LIST | RANK METRIC SCORE | RUNNING ES | CORE ENRICHMENT || 1 | FAM162A | family with sequence similarity 162 member A [Source:HGNC Symbol;Acc:HGNC:17865] | 4 | 1.258 | 0.0711 | Yes |
| 2 | PARL | presenilin associated rhomboid like [Source:HGNC Symbol;Acc:HGNC:18253] | 14 | 1.156 | 0.1359 | Yes |
| 3 | PSMD10 | "proteasome 26S subunit, non-ATPase 10 [Source:HGNC Symbol;Acc:HGNC:9555]" | 58 | 0.976 | 0.1879 | Yes |
| 4 | GHITM | growth hormone inducible transmembrane protein [Source:HGNC Symbol;Acc:HGNC:17281] | 213 | 0.809 | 0.2218 | Yes |
| 5 | FIS1 | "fission, mitochondrial 1 [Source:HGNC Symbol;Acc:HGNC:21689]" | 250 | 0.785 | 0.2635 | Yes |
| 6 | MFF | mitochondrial fission factor [Source:HGNC Symbol;Acc:HGNC:24858] | 932 | 0.531 | 0.2406 | Yes |
| 7 | PYCARD | PYD and CARD domain containing [Source:HGNC Symbol;Acc:HGNC:16608] | 1164 | 0.484 | 0.2501 | Yes |
| 8 | AKT1 | AKT serine/threonine kinase 1 [Source:HGNC Symbol;Acc:HGNC:391] | 1192 | 0.478 | 0.2751 | Yes |
| 9 | MOAP1 | modulator of apoptosis 1 [Source:HGNC Symbol;Acc:HGNC:16658] | 1215 | 0.473 | 0.3002 | Yes |
| 10 | TNFSF10 | TNF superfamily member 10 [Source:HGNC Symbol;Acc:HGNC:11925] | 1241 | 0.468 | 0.3248 | Yes |
| 11 | GPER1 | G protein-coupled estrogen receptor 1 [Source:HGNC Symbol;Acc:HGNC:4485] | 1677 | 0.402 | 0.3138 | Yes |
| 12 | PRKN | parkin RBR E3 ubiquitin protein ligase [Source:HGNC Symbol;Acc:HGNC:8607] | 1972 | 0.364 | 0.3115 | Yes |
| 13 | CLU | clusterin [Source:HGNC Symbol;Acc:HGNC:2095] | 2083 | 0.352 | 0.3229 | Yes |
| 14 | PINK1 | PTEN induced kinase 1 [Source:HGNC Symbol;Acc:HGNC:14581] | 2097 | 0.350 | 0.3417 | Yes |
| 15 | TRIAP1 | TP53 regulated inhibitor of apoptosis 1 [Source:HGNC Symbol;Acc:HGNC:26937] | 2163 | 0.342 | 0.3561 | Yes |
| 16 | IGF1 | insulin like growth factor 1 [Source:HGNC Symbol;Acc:HGNC:5464] | 2286 | 0.328 | 0.3652 | Yes |
| 17 | HGF | hepatocyte growth factor [Source:HGNC Symbol;Acc:HGNC:4893] | 2547 | 0.297 | 0.3618 | No |
| 18 | BIK | BCL2 interacting killer [Source:HGNC Symbol;Acc:HGNC:1051] | 3057 | 0.244 | 0.3361 | No |
| 19 | BAD | BCL2 associated agonist of cell death [Source:HGNC Symbol;Acc:HGNC:936] | 3788 | 0.178 | 0.2894 | No |
| 20 | IFI6 | interferon alpha inducible protein 6 [Source:HGNC Symbol;Acc:HGNC:4054] | 3812 | 0.175 | 0.2975 | No |
| 21 | CIDEB | cell death inducing DFFA like effector b [Source:HGNC Symbol;Acc:HGNC:1977] | 3992 | 0.157 | 0.2925 | No |
| 22 | PLSCR3 | phospholipid scramblase 3 [Source:HGNC Symbol;Acc:HGNC:16495] | 4557 | 0.109 | 0.2547 | No |
| 23 | BNIP3 | BCL2 interacting protein 3 [Source:HGNC Symbol;Acc:HGNC:1084] | 5501 | 0.032 | 0.1832 | No |
| 24 | TP53 | tumor protein p53 [Source:HGNC Symbol;Acc:HGNC:11998] | 5511 | 0.032 | 0.1843 | No |
| 25 | BCL2 | BCL2 apoptosis regulator [Source:HGNC Symbol;Acc:HGNC:990] | 5609 | 0.023 | 0.1780 | No |
| 26 | BCL2L2 | BCL2 like 2 [Source:HGNC Symbol;Acc:HGNC:995] | 5643 | 0.020 | 0.1766 | No |
| 27 | BID | BH3 interacting domain death agonist [Source:HGNC Symbol;Acc:HGNC:1050] | 5715 | 0.014 | 0.1719 | No |
| 28 | PLAUR | "plasminogen activator, urokinase receptor [Source:HGNC Symbol;Acc:HGNC:9053]" | 6362 | -0.032 | 0.1235 | No |
| 29 | BCL2L1 | BCL2 like 1 [Source:HGNC Symbol;Acc:HGNC:992] | 6698 | -0.055 | 0.1005 | No |
| 30 | FZD9 | frizzled class receptor 9 [Source:HGNC Symbol;Acc:HGNC:4047] | 6812 | -0.064 | 0.0954 | No |
| 31 | MLLT11 | MLLT11 transcription factor 7 cofactor [Source:HGNC Symbol;Acc:HGNC:16997] | 7130 | -0.091 | 0.0758 | No |
| 32 | AVP | arginine vasopressin [Source:HGNC Symbol;Acc:HGNC:894] | 7201 | -0.096 | 0.0758 | No |
| 33 | ARRB2 | arrestin beta 2 [Source:HGNC Symbol;Acc:HGNC:712] | 7255 | -0.100 | 0.0773 | No |
| 34 | NOL3 | nucleolar protein 3 [Source:HGNC Symbol;Acc:HGNC:7869] | 7545 | -0.122 | 0.0618 | No |
| 35 | FXN | frataxin [Source:HGNC Symbol;Acc:HGNC:3951] | 8057 | -0.160 | 0.0311 | No |
| 36 | SFN | stratifin [Source:HGNC Symbol;Acc:HGNC:10773] | 8118 | -0.164 | 0.0357 | No |
| 37 | DNM1L | dynamin 1 like [Source:HGNC Symbol;Acc:HGNC:2973] | 8499 | -0.194 | 0.0171 | No |
| 38 | OPA1 | OPA1 mitochondrial dynamin like GTPase [Source:HGNC Symbol;Acc:HGNC:8140] | 8655 | -0.205 | 0.0167 | No |
| 39 | MMP9 | matrix metallopeptidase 9 [Source:HGNC Symbol;Acc:HGNC:7176] | 8698 | -0.209 | 0.0252 | No |
| 40 | TIMM50 | translocase of inner mitochondrial membrane 50 [Source:HGNC Symbol;Acc:HGNC:23656] | 8731 | -0.211 | 0.0347 | No |
| 41 | PDCD5 | programmed cell death 5 [Source:HGNC Symbol;Acc:HGNC:8764] | 9039 | -0.235 | 0.0242 | No |
| 42 | BAK1 | BCL2 antagonist/killer 1 [Source:HGNC Symbol;Acc:HGNC:949] | 9266 | -0.253 | 0.0209 | No |
| 43 | BAX | "BCL2 associated X, apoptosis regulator [Source:HGNC Symbol;Acc:HGNC:959]" | 9323 | -0.257 | 0.0311 | No |
| 44 | PPIF | peptidylprolyl isomerase F [Source:HGNC Symbol;Acc:HGNC:9259] | 9999 | -0.321 | -0.0032 | No |
| 45 | PMAIP1 | phorbol-12-myristate-13-acetate-induced protein 1 [Source:HGNC Symbol;Acc:HGNC:9108] | 10084 | -0.329 | 0.0089 | No |
| 46 | HRK | "harakiri, BCL2 interacting protein [Source:HGNC Symbol;Acc:HGNC:5185]" | 10383 | -0.359 | 0.0061 | No |
| 47 | GGCT | gamma-glutamylcyclotransferase [Source:HGNC Symbol;Acc:HGNC:21705] | 10515 | -0.375 | 0.0172 | No |
| 48 | LMNA | lamin A/C [Source:HGNC Symbol;Acc:HGNC:6636] | 10946 | -0.424 | 0.0078 | No |
| 49 | PLA2G6 | phospholipase A2 group VI [Source:HGNC Symbol;Acc:HGNC:9039] | 11128 | -0.446 | 0.0190 | No |
| 50 | BCL2L11 | BCL2 like 11 [Source:HGNC Symbol;Acc:HGNC:994] | 11867 | -0.564 | -0.0065 | No |
| 51 | BBC3 | BCL2 binding component 3 [Source:HGNC Symbol;Acc:HGNC:17868] | 12298 | -0.676 | -0.0016 | No |
| 52 | SOD2 | superoxide dismutase 2 [Source:HGNC Symbol;Acc:HGNC:11180] | 12685 | -0.853 | 0.0167 | No |
Table: GSEA details [plain text format]

  

Fig 2: GOBP\_RELEASE\_OF\_CYTOCHROME\_C\_FROM\_MITOCHONDRIA      
 Blue-Pink O' Gram in the Space of the Analyzed GeneSet

  

Fig 3: GOBP\_RELEASE\_OF\_CYTOCHROME\_C\_FROM\_MITOCHONDRIA: Random ES distribution      
 Gene set null distribution of ES for **GOBP\_RELEASE\_OF\_CYTOCHROME\_C\_FROM\_MITOCHONDRIA**
